# Transitivity, coherence, and reliability of network meta-analyses comparing proximal humerus fracture treatments: a meta-epidemiological study

**DOI:** 10.1186/s12891-023-07119-w

**Published:** 2024-01-02

**Authors:** Nicolai Sandau, Thomas Vedste Aagaard, Asbjørn Hróbjartsson, Ian A. Harris, Stig Brorson

**Affiliations:** 1 Centre for Evidence-Based Orthopedics, Department of Orthopedic Surgery, Zealand University Hospital, Køge, Denmark; 2 The Research and Implementation Unit PROgrez, Department of Physiotherapy and Occupational Therapy, Naestved-Slagelse-Ringsted Hospitals, Naestved, Denmark; 3 The Department of Regional Health Research, University of Southern Denmark, Odense, Denmark; 4 Centre for Evidence-Based Medicine Odense (CEBMO), and Cochrane Denmark, Department of Clinical Research, University of Southern Denmark, Odense, Denmark; 5 Open Patient data Explorative Network (OPEN), Odense University Hospital, Odense, Denmark; 6 Whitlam Orthopaedic Research Centre, Ingham Institute for Applied Medical Research, South Western Sydney Clinical School, University of New South Wales (UNSW Sydney), Liverpool, NSW Australia

**Keywords:** Proximal humerus fractures, Shoulder fractures, Network meta-analyses, Methodological quality, Transitivity, Indirectness, Coherence, Consistency, Confidence in results

## Abstract

**Background:**

Network meta-analyses can be valuable for decision-makers in guiding clinical practice. However, for network meta-analysis results to be reliable, the assumptions of both transitivity and coherence must be met, and the methodology should adhere to current best practices. We aimed to assess whether network meta-analyses of randomized controlled trials (RCTs) comparing interventions for proximal humerus fractures provide reliable estimates of intervention effects.

**Methods:**

We searched PubMed, EMBASE, The Cochrane Library, and Web of Science for network meta-analyses comparing interventions for proximal humerus fractures. We critically assessed the methodology regarding the development of a protocol, search strategy, trial inclusion, outcome extraction, and the methods used to conduct the network meta-analyses. We assessed the transitivity and coherence of the network graphs for the Constant score (CS), Disabilities of the Arm, Shoulder, and Hand score (DASH), and additional surgery. Transitivity was assessed by comparing probable effect modifiers (age, gender, fracture morphology, and comorbidities) across intervention comparisons. Coherence was assessed using Separating Indirect from Direct Evidence (SIDE) (Separating Indirect from Direct Evidence) and the design-by-treatment interaction test. We used CINeMA (Confidence in Network Meta-analyses) to assess the confidence in the results.

**Results:**

None of the three included network meta-analyses had a publicly available protocol or data-analysis plan, and they all had methodological flaws that could threaten the validity of their results. Although we did not detect incoherence for most comparisons, the transitivity assumption was violated for CS, DASH, and additional surgery in all three network meta-analyses. Additionally, the confidence in the results was ‘very low’ primarily due to within-study bias, reporting bias, intransitivity, imprecision, and heterogeneity.

**Conclusions:**

Current network meta-analyses of RCTs comparing interventions for proximal humerus fractures do not provide reliable estimates of intervention effects. We advise caution in using these network meta-analyses to guide clinical practice. To improve the utility of network meta-analyses to guide clinical practice, journal editors should require that network meta-analyses are done according to a predefined analysis plan in a publicly available protocol and that both coherence and transitivity have been adequately assessed and reported.

**Supplementary Information:**

The online version contains supplementary material available at 10.1186/s12891-023-07119-w.

## Background

Network meta-analyses can be a valuable tool for decision-makers in guiding clinical practice and have seen wide adoption in many clinical areas [1]. Compared to standard pairwise meta-analyses that only use direct comparisons between two interventions, network meta-analyses can incorporate indirect comparisons between interventions if the compared interventions have a common comparator [2]. Consequently, network meta-analyses can compare more than two interventions, and the additional indirect evidence may lead to increased precision. However, the validity of the results obtained using indirect comparisons relies on certain core assumptions.

The main assumptions of network meta-analyses are the assumptions of *transitivity* and *coherence*, where coherence is the statistical equivalent of transitivity [2]. Practically, transitivity means that one should be able to conduct one multi-arm RCT with all interventions of interest. Thus, any probable effect modifiers would then be similar between interventions. An example of when the transitivity assumption could be violated is if intervention A is primarily administered to younger patients while intervention B is primarily administered to older patients. If age is a probable effect modifier, the transitivity assumption will be violated (*intransitivity*). Coherence is when the effect estimates obtained through direct and indirect comparisons agrees [2]. In the example above, the lack of transitivity could lead to spurious effect estimates for the indirect comparisons causing the effect estimates obtained through direct and indirect comparisons to differ. This difference between estimates would violate the coherence assumption (*incoherence*).

The Grading of Recommendations and Assessment, Development, and Evaluation (GRADE) framework is widely used to assess the confidence in the results for meta-analyses of standard pairwise comparisons. Recently, a new framework, Confidence in Network Meta-analyses (CINeMA), was introduced [3]. CINeMA is broadly based on GRADE but has been adapted to network meta-analyses. The authors of CINeMA have developed an online web application where authors can assess the confidence in the results based on the evaluations of six domains: *within-study bias*, *reporting bias*, *indirectness*, *imprecision*, *heterogeneity*, and *incoherence*.

Many trials have compared interventions for proximal humerus fractures, but consensus about the optimal treatments is still lacking. In a previous study, we identified two network meta-analyses comparing interventions for the treatment of proximal humerus fractures, which both concluded in favor of reverse shoulder arthroplasty (RSA) compared to open reduction and internal fixation (ORIF), intramedullary nail (IMN), hemi-arthroplasty (HA), and nonoperative (NOP) treatment [4–6]. However, the authors did not report an assessment of transitivity, nor did they use a structured framework to assess the confidence in the results.

We aim to assess whether network meta-analyses of RCTs comparing interventions for proximal humerus fractures provide reliable estimates of intervention effects.

## Methods

The study was registered on August 12, 2022, and the protocol is available at https://osf.io/x5b64.

### Search

We searched PubMed, EMBASE, The Cochrane Library, and Web of Science from inception to August 12, 2022, for network meta-analyses comparing interventions for proximal humerus fractures. The search strategy is available in Appendix A (Table A.1). The titles and abstracts of the obtained records were screened for potential eligibility in duplicate by NS and TVA. Full texts were obtained for the potentially eligible records and screened for final inclusion. No limitations were set for the publication date or language. Any disagreements were resolved by consensus.

We excluded network meta-analyses that included non-randomized trials. This was done because we have previously reported that only one of 16 non-randomized trials included in meta-analyses comparing operative with NOP interventions for proximal humerus fractures reported outcomes adjusted for confounding [7]. Including such unadjusted outcomes in network meta-analyses increase the risk of reporting spurious results, and, in general, including non-randomized trials in a network meta-analysis is not recommended [2].

### Data extraction

From the included network meta-analyses, we extracted the following: 1) the rationale for conducting the network meta-analysis, 2) whether previous network meta-analyses were referenced, 3) whether a predefined protocol or data analysis plan was publicly available, 4) the Population, Intervention, Comparator, Outcome (PICO) criteria, 5) whether grey literature was searched, 6) whether, and how, the transitivity and coherence assumptions were assessed, 7) whether the authors included a statistician with experience in network meta-analyses, 8) the statistical framework (Bayesian or frequentist) and software used to conduct the network meta-analysis, and 9) the RCTs from which outcome data were used for the analyses.

We emailed the corresponding authors requesting a copy of their extracted outcome data used to conduct their analyses, the code used to conduct the network meta-analyses, and their protocol or data analysis plan. We also inquired whether one or more authors had experience conducting or interpreting network meta-analyses and how they handled missing standard deviations (SDs) for continuous outcomes.

From each identified RCT, we extracted the reported PICO criteria, the mean age of participants, the proportion of females included as participants, fracture classifications, and whether the trial was either publicly registered or a protocol had been published. If a trial registration or protocol was available, we also noted whether there were any discrepancies between the planned outcome measures and the outcome measures reported in the trial report.

To assess the confidence in the results using the CINeMA web application, we had to obtain outcome data and risk of bias assessments for each of the identified RCTs [3]. We extracted results for the following outcomes: Constant score (CS), Disabilities of the Arm, Shoulder, and Hand score (DASH), and additional surgery (defined as surgical revision or secondary surgery). Following our protocol, we did not obtain outcomes for the Oxford shoulder score because none of the network meta-analyses included this outcome in their analyses. Only one of the network meta-analyses (Du 2017) reported the outcomes extracted from their included trials, and this was only for the CS reported in a subset of the identified RCTs. We, therefore, primarily extracted outcome data from a recent Cochrane Review comparing operative with NOP interventions for proximal humerus fractures coauthored by SB [8]. For the RCTs not included in the Cochrane review, the outcome data were extracted directly from the trial report. The extraction of outcome data was performed in duplicate by NS and TVA using a piloted spreadsheet, with disagreements resolved by consensus.

The risk of bias assessments was also extracted from the aforementioned Cochrane review coauthored by SB [8]. These risk of bias assessments were performed using the Cochrane Risk of Bias tool (Version 1). For the RCTs not included in the Cochrane review, NS and THA performed a risk of bias assessment in duplicate using the Cochrane Risk of Bias tool (version 1). Based on these assessments, an overall risk of bias of either ‘low’, ‘moderate’, or ‘high’ was assigned to each RCT [9]. Disagreements were resolved by consensus.

### Data analysis

#### Critical appraisal of the methodology

We critically appraised the methodology concerning the development of a protocol, search strategy, trial inclusion, outcome extraction, and the methods used to conduct the network meta-analyses. We based our assessments on the best practices reported in the Cochrane Handbook for Systematic Reviews of Interventions [10].

#### Assessment of transitivity

For each network meta-analysis, we assessed the transitivity of the included RCTs by comparing probable effect modifiers across intervention comparisons. We focused on effect modifiers which have been identified as probable predictors of outcome in patients with proximal humerus fractures: age, gender, fracture morphology, and comorbidities [11–19]. We did this by qualitatively comparing the reported PICO criteria and quantitatively comparing the overall mean age and proportion of included females across intervention comparisons. It was not possible to quantitatively compare comorbidities and fracture morphology due to heterogeneity in both the classification and reporting between trials.

For each of the intervention comparisons, the mean ages reported by the trials comprising these comparisons were combined into one overall mean using the formula described in the Cochrane Handbook for Systematic Reviews of Interventions (Table 6.5.a) [10]. Similarly, the overall proportion of females included in each of the trials was calculated for each of the intervention comparisons.

For both the qualitative and quantitative assessments of transitivity, we used the comparison between HA and NOP as a reference because all the included network meta-analyses included the same trials for this comparison and because the populations for these two trials were very similar [20, 21].

In our protocol, we estimated that a statistically significant absolute difference over 0.20 in the overall proportion of females and over 5 years for the overall mean age of participants to potentially have a clinically meaningful effect on the outcomes, thereby indicating a lack of transitivity. As these cutoffs are based on our best estimates, we also conducted sensitivity analyses using different cutoffs, as described in section 2.4. For each of the intervention comparisons, we used Fisher’s exact test to determine if the differences in the overall proportion of females were statistically significant compared to the reference comparison. We planned to use the unpaired two-sample t-test to compare whether the overall mean age of participants for each intervention comparison differed significantly from the reference comparison, but many of the identified RCTs did not report an SD. Therefore, we could not perform the planned tests for the mean age. Consequently, we used the previously defined absolute difference in age as an indication of intransitivity.

Based on these qualitative and quantitative assessments, we determined whether there was intransitivity between each of the intervention comparisons. The assessment of transitivity was conducted in duplicate by NS and TVA, with disagreements resolved by consensus.

#### Assessment of coherence

The incoherence of the network meta-analyses was assessed using two different methods: the SIDE test and the design-by-treatment interaction test [22–24]. The SIDE method calculates the effect estimates obtained using only direct and indirect evidence and tests whether these estimates are statistically different. However, SIDE cannot be used for comparisons that rely on only direct or indirect evidence [22]. In such situations, we will use the design-by-treatment interaction test, a global test that estimates the incoherence of effect estimates between intervention comparisons [24]. Both methods have low power and can, therefore, only be used to detect incoherence, not as evidence for coherence [2]. For this reason, we set a *p*-value less than 0.10 to indicate incoherence.

To conduct the aforementioned analyses of coherence, we performed a network meta-analysis. However, we have intentionally not reported any of the obtained effect estimates from the meta-analyses, as this was not the aim of our study.

#### Assessment of the confidence in the results

The confidence in the results was assessed using the CINeMA web application [3, 25]. A more detailed description of the CINeMA tool can be found in our protocol and the primary papers by the authors of CINeMA [3, 25].

In short, the CINeMA web application assesses six domains (within-study bias, reporting bias, indirectness, imprecision, heterogeneity, and incoherence) for each intervention comparison and then assigns a level of concern (no concerns, some concerns, major concerns). To achieve this, the tool conducts a network meta-analysis and calculates the contribution of each included trial to the obtained results. The tool does not report any effect estimates derived from these network meta-analyses. The CINeMA tool uses indirectness and directness for intransitivity and transitivity, respectively. For consistency throughout this paper, we will continue to use the latter terms.

Based on the assessments for the six domains, each intervention comparison is assigned an overall level of confidence in the result (‘very low’, ‘low’, ‘moderate’, or ‘high’). Similar to GRADE, all comparisons start at ‘high’ and are then downgraded a step for each domain rated as ‘some concerns’, and two steps for each domain rated as ‘major concerns ’[3].

To perform the assessments of the six domains, the CINeMA web application requires the following information for each outcome of interest: trial level outcome data, risk of bias assessments for each trial, a minimal clinically important difference, an assessment of the risk of reporting bias for each pairwise comparison, and an assessment of transitivity.

In addition, one has to decide on a summarization rule specifying how the contributions of each trial should be weighted for the within-study bias and intransitivity domains. We chose the weighted average rule such that the assessments are weighted by the percentage of contribution to the estimate for the given intervention comparison.

Reporting bias was assessed using the indicators provided in the CINeMA publication: 1) a failure to include unpublished data and data from grey literature, 2) the meta-analysis is based on a small number of positive early findings, 3) the intervention comparison is studied exclusively or primarily in industry-funded trials, and 4) there is previous evidence documenting the presence of reporting bias [3]. Reporting bias was also suspected if one or more trials within a given intervention comparison had discrepancies between the reported outcome measures and the trial registration or if no trial or protocol registration pre-dating the start of the trial was available.

Each RCT was assigned a level of intransitivity of either ‘low’, ‘moderate’, or ‘high’, based on the aforementioned transitivity assessments. An RCT was determined to have ‘moderate’ intransitivity if one probable effect modifier was assessed as lacking transitivity (i.e., age difference larger than 5 years), and a rating of ‘high’ if it was more than one.

As described in our protocol, the minimal clinically important difference was set to 5.4 for CS and 8.1 for DASH [26, 27]. For additional surgery, we determined that any increase in risk would be clinically relevant.

### Sensitivity analyses

Per our protocol, we conducted a sensitivity analysis using a difference of 0.30 for the overall proportion of included females and 10 years for the overall mean age of the included participants.

We also conducted two post-hoc sensitivity analyses. In the first, we imputed the SDs for any trials with missing SDs, and then reperformed our analyses with those trials included. The SDs were imputed by using the mean SD for the outcome based on the studies included in that respective network meta-analysis. In the second, we performed our analyses for the network meta-analyses we had to exclude due to the inclusion of non-randomized studies, but we only used their included RCTs.

## Results

### Search

The search returned 2210 records, from which we included 3 network meta-analyses, which we will refer to as Davey 2021 [28], Orman 2020 [29], and Du 2017 [5]. We excluded another network meta-analysis due to including non-randomized studies (Chen 2016) [4]. A PRISMA flowchart is available in Appendix A (Fig. A.1).

### Characteristics

The characteristics of the included network meta-analyses are presented in Table 1. Notably, none of the network meta-analyses reported assessing the transitivity of the network meta-analyses. However, all three network meta-analyses assessed the coherence, but only Du 2017 reported the results.

**Table 1:** Study characteristics of the included network meta-analyses

NMA	Population	Interventions	Rationale	Protocol available	NMA type (software)	Transitivity / coherence assessment
Davey 2021	“Patients included in RCTs who have undergone management of proximal humerus fractures.”	NOP, LCP, IMN, HA, RSA.	“(...) many new randomized control trials have since been published on the topic, an updated systematic review and network meta-analysis which focuses on outcomes of all displaced proximal humerus fractures, including analysis of IMN is warranted.”	No	Frequentist (RevMan and netmeta package in R).	None / I^2^-index
Du 2017	“3- or 4-part proximal humeral fractures in senile patients”	NOP, HA, RSA and ORIF (not defined further, but trials with LCP and TB included as ORIF).	“(...) there is no RCTs to evaluate the clinical outcomes after conservative treatment and RSA to date. Therefore, it seems to be particularly important that more high-level evidence-based medical researches are expected to evaluate the value of the therapies.”	No	Bayesian (rjags and gemtc packages in R).	None / Node-splitting
Orman 2020	“3-part or 4-part proximal humerus fractures”	NOP, HA, RSA, and ORIF (defined as LCP, but authors also include TB as ORIF)	“(...) previous network meta-analyses have resorted to including non-RCT studies as well as using wide age ranges, which may have diluted the reliability of their findings.”	No	Frequentist (Comprehensive Meta-Analysis version 2)	None / I^2^-index

*NMA* Network meta-analysis, *RCT* Randomized controlled trial, *HA* Hemi-arthroplasty, *NOP* Nonoperative, *LCP* Locking compression plate, *IMN* Intramedullary nail, *RSA* Reverse shoulder arthroplasty, *TB* Tension-band

Both Davey 2021 and Orman 2020 referenced previously published network meta-analyses [4, 5]. None of the network meta-analyses reported searching grey literature. It was unclear from the information reported in the network meta-analyses whether one of the authors was a statistician with experience conducting and interpreting network meta-analyses.

All of the network meta-analyses included outcomes for locking compression plate (LCP), RSA, HA, and NOP. Du 2017 and Orman 2020 also included outcomes for tension-band (combined with LCP as ORIF), while Davey 2021 also included outcomes for IMN.

Du 2017 included 7 trials, all of which were also included in Orman 2020 [20, 21, 30–34]. Orman 2020 included an additional trial not included in the two other network meta-analyses [35]. In contrast to the two other network meta-analyses, Davey 2021 included an additional 6 trials but did not include the trial by Zyto et al. [36–41]. The characteristics of the trials included in the three network meta-analyses are available in Appendix A (Table A.2).

None of the corresponding authors responded to our inquiry regarding their protocol or data analysis plan, the extracted outcome data and code used to conduct their analyses, how missing data was handled, and whether one or more authors had experience conducting or interpreting network meta-analyses.

### Critical appraisal of the methodology

#### Protocol

None of the three network meta-analyses had a publicly available protocol or data analysis plan. As we have previously reported, meta-analyses without a protocol have high analytical flexibility, potentially allowing for data-contingent decisions which may threaten the validity of the obtained results [7].

#### Trial inclusion

In Davey 2021, the authors reported including a trial named ‘Leighton et al.’, which is not included in the two other network meta-analyses [42]. However, the referenced paper is a commentary regarding a trial by Olerud et al., which is also included in Davey 2021. Therefore, the results for CS and additional surgery mentioned in the commentary are the results obtained by Olerud and colleagues [32]. Consequently, including the results from ‘Leighton et al.’ leads to double-counting of the results reported by Olerud et al., thereby causing a spurious increase in the precision of the effect estimates. In the following assessments of transitivity, coherence, and confidence in the results, we will not include the results for CS or additional surgery reported in the ‘Leighton et al.’ reference.

In their inclusion criteria, the authors of Orman 2020 reported ORIF with LCP as an intervention of interest. However, the authors included the trial by Zyto et al. with the surgical intervention classified as ORIF, although that trial compared tension-band with NOP [34]. Their results for the CS are, therefore, not generalizable to their population of interest and should be interpreted as such. The authors of Orman 2020 did not report why the trial by Zyto et al. was included as part of the ORIF intervention group.

#### Outcome extraction

All three network meta-analyses included outcomes from the trial by Cai et al. [30]. However, the results for both CS and DASH are reported without an SD. To use these results in their analyses, the SD must have either been obtained directly from the trial authors or by imputation. Du 2017 reports an SD of 12.3 for the CS outcome obtained from Cai et al. This is the SD obtained by imputation based on the reported *p*-values when assuming that a t-test was used. However, Cai et al. report using a non-parametric test (Mann-Whitney U), meaning that imputation using the p-value is problematic. The authors of Davey 2021 and Orman 2020 did not report how the SDs were obtained, nor did they report the extracted outcomes used in their analyses. As we were unable to obtain a valid SD, we did not include the results from Cai et al. when assessing coherence and confidence in the results for CS and DASH.

The authors of Orman 2020 included outcomes from the trial by Chen et al. in their analyses [35]. However, Chen et al. reported both CS and DASH as relative scores in percent compared to the unaffected shoulder [35]. Such relative scores are less robust than absolute scores, given that the reported results are now dependent on the functional outcome of the unaffected shoulder. Therefore, the results of both standard pairwise and network meta-analyses that include such relative scores should be interpreted with caution.

#### Statistical methods

The authors of Orman 2020 reported using the software ‘Comprehensive Meta-analysis’ for their statistical analyses. However, we have inquired with Biostat Inc., the company behind ‘Comprehensive Meta-analysis’, and they informed us that their software does not perform network meta-analyses [43]. We have inquired with the corresponding author how they performed the network meta-analyses using the aforementioned software, but we have not received a response.

### Transitivity and coherence

**Fig. 1:**
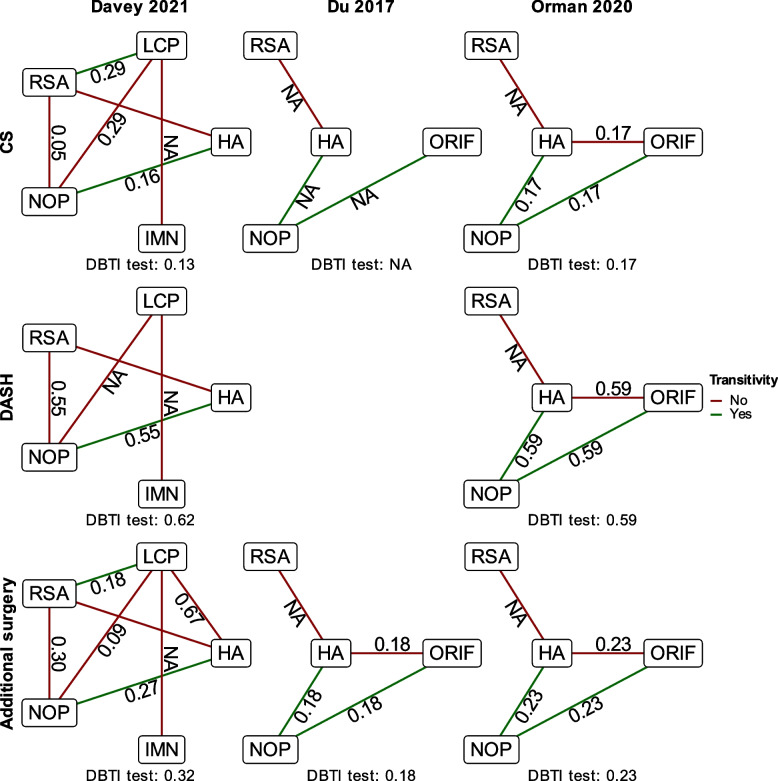
Network graphs with transitivity and coherence for the included network meta-analyses. The numbers along the graph lines are the *p*-values obtained using SIDE (Separating Indirect from Direct Evidence) for that comparison. DBTI: Design-by-treatment interaction, NA: not applicable due to lack of closed loop

The network graphs with transitivity and coherence results for the three network meta-analyses are shown in Fig. 1. Notably, without the trial by Cai et al., the network for the CS in Du 2017 has no closed loops and can, therefore, not utilize indirect comparisons [30].

One trial which only included fractures with an absolute surgery indication provided evidence for the RSA:HA comparison [33]. We, therefore, assessed the RSA:HA comparison for all three network meta-analyses as lacking transitivity because the trial by Olerud et al. (comparing HA with NOP) specifically excluded such fractures [21, 33].

The results of our quantitative assessments of transitivity for the overall mean age and gender distribution of trial participants are presented in Table 2. Besides the results, it is also worth noting that many comparisons only consist of one trial and that the highest number of trials for any comparison is only three. The networks for all three network meta-analyses are, therefore, very sparse.

**Table 2:** Quantitative assessment of transitivity for mean age and gender of trial participants

Comparison	RCTs	Participants	Age, years		Females, %	
			Mean (SD)	Difference	Included	Difference
**Davey 2021**						
HA:NOP	2	105	77.4 (NA)	*reference*	90	*reference*
LCP:HA	1	32	71.6 (NA)	−5.7	84	−6 (ns)
LCP:IMN	3	184	65.0 (NA)	−12.3	72	− 18 (*)
LCP:NOP	3	197	73.0 (NA)	−4.4	87	−3 (ns)
LCP:RSA	1	124	75.2 (6.4)	−2.2	90	0 (ns)
RSA:HA	1	61	74.0 (NA)	−3.4	85	−5 (ns)
RSA:NOP	1	59	83.5 (5.1)	6.2	86	−4 (ns)
**Du 2017**						
HA:NOP	2	105	77.4 (NA)	*reference*	90	*reference*
ORIF:HA	1	32	71.6 (NA)	−5.7	84	−6 (ns)
ORIF:NOP	3	149	73.5 (NA)	−3.9	85	−5 (ns)
RSA:HA	1	61	74.0 (NA)	−3.4	85	−5 (ns)
**Orman 2020**						
HA:NOP	2	105	77.4 (NA)	*reference*	90	*reference*
ORIF:HA	2	92	68.0 (NA)	−9.4	64	−26 (*)
ORIF:NOP	3	149	73.5 (NA)	−3.9	85	−5 (ns)
RSA:HA	1	61	74.0 (NA)	−3.4	85	−5 (ns)

*: *p* < 0.001, ns: not significant, *HA* Hemi-arthroplasty, *NOP* Nonoperative, *LCP* Locking compression plate, *IMN* Intramedullary nail, *RSA* Reverse shoulder arthroplasty, *ORIF* Open reduction internal fixation, *SD* Standard deviation, *NA* Not available due to some studies not reporting standard deviations

#### Davey 2021

For Davey 2021, we assessed the comparisons LCP:HA, LCP:IMN, and RSA:NOP as lacking transitivity due to an absolute difference larger than 5 years in the mean age of the participants (Table 2). The LCP:IMN comparison also included significantly fewer female participants, but the difference was less than our predefined cutoff of an absolute difference larger than 20% (Table 2). We also assessed the LCP:NOP comparison as lacking transitivity due to the trial by Launonen et al. only including 2-part fractures while constituting 45% of the total sample size for that comparison (Table A.2) [38]. The SIDE test showed signs of incoherence for the RSA:NOP comparison in the network for CS (*p* = 0.05), and for the NOP:LCP comparison in the network for additional surgery (*p* = 0.09) (Fig. 1).

#### Du 2017

For Du 2017, we determined the ORIF:HA comparison as lacking transitivity due to a younger population compared to the reference population (Table 2). Due to the network for the CS not having closed loops, we could not calculate the coherence for the CS. The coherence tests for additional surgery did not find evidence of incoherence for any comparisons.

#### Orman 2020

For Orman 2020, the comparison for ORIF:HA was assessed as lacking transitivity due to a younger population consisting of significantly fewer females compared to the reference comparison (Table 2). Neither the global nor the SIDE tests found evidence of incoherence for any of the three outcome domains.

### Confidence in the results

The reporting bias used in the CINeMA web application was set to ‘some concern’ for all comparisons due to the many trials without a trial registration combined with a relatively high rate of discrepancy between planned and reported trial outcomes for the trials with a registration (Table A.2). In addition, none of the network meta-analyses searched for grey literature.

Using the CINeMA web application, all comparisons for all outcome domains in all three network meta-analyses were rated as ‘very low’ confidence in the results. A summary of the reasons for downgrading is shown in Table 3. Notably, all comparisons for all three network meta-analyses were downgraded due to issues with within-study bias and reporting bias. However, most comparisons were also downgraded due to intransitivity, imprecision, and heterogeneity issues. Only a few of the comparisons were downgraded due to issues with incoherence. The CINeMA tool could not calculate the imprecision, heterogeneity, and incoherence for DASH in Orman 2020 due to a sparse network of only 1 trial per comparison without closed loops.

**Table 3:** Reasons for downgrading the confidence in the results

Outcome domains	Studies, N	Comparisons, N	Within-study bias, %	Reporting bias, %	Intransitivity, %	Imprecision, %	Heterogeneity, %	Incoherence, %
**Davey 2021**								
CS	11	10	100	100	60	50	30	10
DASH	7	10	100	100	90	90	70	0
Additional surgery	11	10	100	100	40	60	30	10
**Du 2017**								
CS	6	6	100	100	33	50	50	100
Additional surgery	6	6	100	100	67	67	33	0
**Orman 2020**								
CS	7	6	100	100	67	50	67	0
DASH	4	6	100	100	83	NA	NA	NA
Additional surgery	7	6	100	100	67	67	33	0

*CS* Constant score, *DASH* Disabilities of the Arm, Shoulder and Hand, *NA* Not applicable

### Sensitivity analyses

When using a difference of 0.30 for the overall proportion of included females and 10 years for the overall mean age of the included participants, the transitivity assessments were mostly unchanged. For Davey 2021, only the RSA:NOP comparison for all three outcome domains and the LCP:HA comparison for additional surgery were no longer assessed as lacking transitivity. For Orman 2020, only ORIF:HA for the additional surgery domain was no longer assessed as lacking transitivity. All comparisons for Du 2017 were unchanged. The network graphs for the sensitivity analysis are available in Appendix A (Fig. A.2). Although the sensitivity analysis reduced the prevalence of intransitivity as a reason for downgrading, the confidence in the results for all comparisons in all three network meta-analyses were still rated as ‘very low’ (Appendix A Table A.3).

When including the imputed results for CS and DASH from Cai et al., the graphs still had issues with intransitivity (Appendix A Fig. A.3) [30]. In addition, for the CS graph in Davey 2021, the SIDE test indicated incoherence for the LCP:HA and RSA:HA comparisons, and the design-by-interaction test was significant. The confidence in the results was still ‘very low’ for all comparisons in all of the three network meta-analyses, and the prevalence of the reasons for downgrading was similar to our primary analysis (Appendix A Table A.4).

The results for the excluded network meta-analysis (Chen 2016) were similar to the three included network meta-analyses [4]. The comparisons of both HA:RSA and ORIF:IMN showed signs of intransitivity. The graph for the CS was sparse and lacked a closed loop, and we could, therefore, not calculate the incoherence (Appendix A Fig. A.4). The confidence in the results was ‘very low’ for all comparisons. The prevalence of the reasons for downgrading was similar to the three included network meta-analyses (Appendix A Table A.5).

## Discussion

We found that none of the three included network meta-analyses had a publicly available protocol or data-analysis plan, and they all had methodological flaws that could threaten the validity of their results. Although we did not detect incoherence for most comparisons, the transitivity assumption was violated for CS, DASH, and additional surgery in all three network meta-analyses. Additionally, the confidence in the results was ‘very low’ primarily due to within-study bias, reporting bias, intransitivity, imprecision, and heterogeneity.

### Comparisons to previous studies

Our finding that none of the network meta-analyses reported assessing transitivity is similar to what has previously been reported. In a previous cross-sectional study, only 23% of network meta-analyses published between 1999 and 2015 reported assessing transitivity [1]. Similarly, a cross-sectional study of network meta-analyses using individual participant data showed that none of the included network meta-analyses assessed transitivity [44]. In studies assessing the quality of reporting for network meta-analyses regarding specific clinical questions, the results are similar, with the prevalence of assessing transitivity ranging from 13 to 35% [45–48].

### Interpretations and implications

That many comparisons lacked transitivity is unsurprising, given that the interventions used for proximal humerus fractures are often offered to differing populations. ORIF is traditionally offered to younger patients with less complex fractures and fewer comorbidities, while older age is associated with a higher likelihood of receiving NOP intervention [49–52]. It may be difficult to combine such distinct populations in a network meta-analysis without violating the transitivity assumption, and doing so may threaten the validity of the results [2].

Given that all comparisons had very low confidence in the results, conclusions such as “(…) RSA is the optimum treatment (…)” [5] and “RSA offers satisfactory improvements in clinical and functional outcomes when compared to other non-operative and operative treatment options (…)” [28] are overinterpretations of the available evidence. We, therefore, advise caution in using the results and conclusions of these network meta-analyses to guide clinical practice and recommend that authors of future network meta-analyses comparing interventions for proximal humerus fractures use a structured tool to assess the confidence in the results.

Notably, many of the methodological flaws (e.g., including the same results twice and reporting using software that does not support such analyses) and transparency issues (e.g., not responding to letters and not making a protocol or data-analysis plan publicly available) we identified are not specific to network meta-analyses. Instead, they are indicators of a general lack of scientific rigor. Such practices can hurt reproducibility and slow scientific progress and may also result in patients receiving interventions based on invalid or biased evidence [53].

Our study highlights an important issue regarding the publication of network meta-analyses not reporting on the assessment of a fundamental assumption underlying their analyses. As previously mentioned, this seems to be a widespread issue across multiple clinical areas. This is concerning, given that the results obtained from network meta-analyses are invalid if the transitivity assumption is violated [54, 55]. Journal editors should therefore require that network meta-analyses adequately assess and report both coherence and transitivity before being eligible for publication.

### Strengths and limitations

The strengths of this study are the use of both quantitative and qualitative assessments of transitivity and a structured tool to assess confidence in the results. The study also has certain limitations. The assessment of transitivity is inherently subjective, and others may therefore obtain differing assessments. However, we have tried to mitigate this by conducting a sensitivity analysis. Additionally, as shown by the sensitivity analysis, even when the prevalence of intransitivity for the comparisons decreased, the confidence in the results remained unchanged due to issues in multiple other areas. Furthermore, due to unmeasured or unreported effect modifiers within the RCTs, the assessment of transitivity may be biased. As a result, some comparisons that were assessed as not violating the transitivity assumption may have done so.

## Conclusions

In conclusion, current network meta-analyses of RCTs comparing interventions for proximal humerus fractures do not provide reliable estimates of intervention effects. We advise caution in using these network meta-analyses to guide clinical practice. To improve the utility of network meta-analyses to guide clinical practice, journal editors should require that network meta-analyses are done according to a predefined analysis plan in a publicly available protocol and that both coherence and transitivity have been adequately assessed and reported.

### Supplementary Information

**Additional file 1:** **Appendix A.**

## Data Availability

The datasets used and/or analysed during the current study are available from the corresponding author on reasonable request.

## Abbreviations

CINeMA: Confidence in network meta-analyses
CS: Constant score
DASH: Disabilities of the arm, shoulder, and hand score
GRADE: Grading of recommendations and assessment, development, and evaluation
HA: Hemi-arthroplasty
IMN: Intramedullary nail
LCP: Locking compression plate
NOP: Nonoperative
ORIF: Open reduction and internal fixation
PICO: Population, intervention, comparator, outcome
RCT: Randomized controlled trial
RSA: Reverse shoulder arthroplasty
SD: Standard deviation
SIDE: Separating indirect from direct evidence
