# Supplementary material for: Transitivity, coherence, and reliability of network meta-analyses comparing proximal humerus fracture treatments: a meta-epidemiological study
Source: BMC Musculoskelet Disord. 2024 Jan 2;25:14. doi: 10.1186/s12891-023-07119-w (PMC10759380; doi:10.1186/s12891-023-07119-w)
Supplement: Supplementary file 1 — Additional file 1: Appendix A. [file 12891_2023_7119_MOESM1_ESM.pdf]

Table of Contents

Table A.1: Search strategy....2

Fig. A.1: PRISMA Flowchart....3

Table A.2: Trial characteristics....5

Fig. A.2: Sensitivity analysis - network graphs....11

Table A.3: Sensitivity analysis - CINeMA reasons for downgrading....11

Fig. A.3: Cai et al. 2012 sensitivity analysis - network graph....12

Table A.4: Cai et al. 2012 sensitivity analysis - CINeMA reasons for downgrading....12

Fig. A.4: Chen 2016 sensitivity analysis - network graph....13

Table A.5: Chen 2016 sensitivity analysis - CINeMA reasons for downgrading....13

Table A.1: Search strategy

Database	Search strategy
PubMed	1. ”Humeral Fractures”[MeSH] OR ”Shoulder Fractures”[MeSH] OR ((Shoulder[MeSH] OR “Shoulder Joint”[MeSH]) AND “Fractures, Bone”[MeSH])<br>2. (Shoulde* AND fract*) OR ((proxim* OR neck* OR sub-capit* OR subcapit*) AND humer* AND fract*)<br>3. #1 OR #2<br>4. "Systematic Review" [Publication Type] OR "Systematic Reviews as Topic"[Mesh] OR "Meta-Analysis" [Publication Type] OR "Meta-Analysis as Topic"[Mesh]<br>5. systemati* OR cochrane OR pubmed OR medline OR embase OR meta-ana* OR metaana*<br>6. #4 OR #5<br>7. #3 AND #6
EMBASE	1. exp Humerus Fracture/ OR exp Shoulder Fracture/ OR (exp Shoulder/ AND exp fracture/)<br>2. ((Shoulde* adj8 fract*) or ((proxim* or neck* or sub-capit* or subcapit*) adj8 humer* adj8 fract*)).mp.<br>3. 1 OR 2<br>4. exp "systematic review"/ OR exp "systematic review (topic)"/ OR exp meta analysis/ OR exp "meta analysis (topic)"/<br>5. (systemati* OR cochrane OR pubmed OR medline OR embase OR meta-ana* OR metaana*).mp.<br>6. 4 OR 5<br>7. 3 AND 6
Cochrane Library	1. [mh "Humeral Fractures"] OR [mh "Shoulder Fractures"] OR (([mh Shoulder] OR [mh "Shoulder Joint"]) AND [mh "Fractures, Bone"])<br>2. (Shoulde* NEAR/8 fract*) OR ((proxim* OR neck* OR sub-capit* OR subcapit*) NEAR/8 humer* NEAR/8 fract*)<br>3. #1 OR #2<br>4. [mh "Systematic Reviews as Topic"] OR [mh "Meta-Analysis as Topic"]<br>5. systemati* OR (cochrane OR pubmed OR medline OR embase):ti,ab OR meta-ana* OR metaana*<br>6. #4 OR #5<br>7. #3 AND #6
Web of Science	1. TI = ((Shoulde* NEAR/8 fract*) OR ((proxim* OR neck* OR sub-capit* OR subcapit*) NEAR/8 humer* NEAR/8 fract*)) OR AB = ((Shoulde* NEAR/8 fract*) OR ((proxim* OR neck* OR sub-capit* OR subcapit*) NEAR/8 humer* NEAR/8 fract*))<br>2. TI = (systemati* OR cochrane OR pubmed OR medline OR embase OR meta-ana* OR metaana*) OR AB = (systemati* OR cochrane OR pubmed OR medline OR embase OR meta-ana* OR metaana*)<br>3. #1 AND #2

Fig. A.1: PRISMA Flowchart

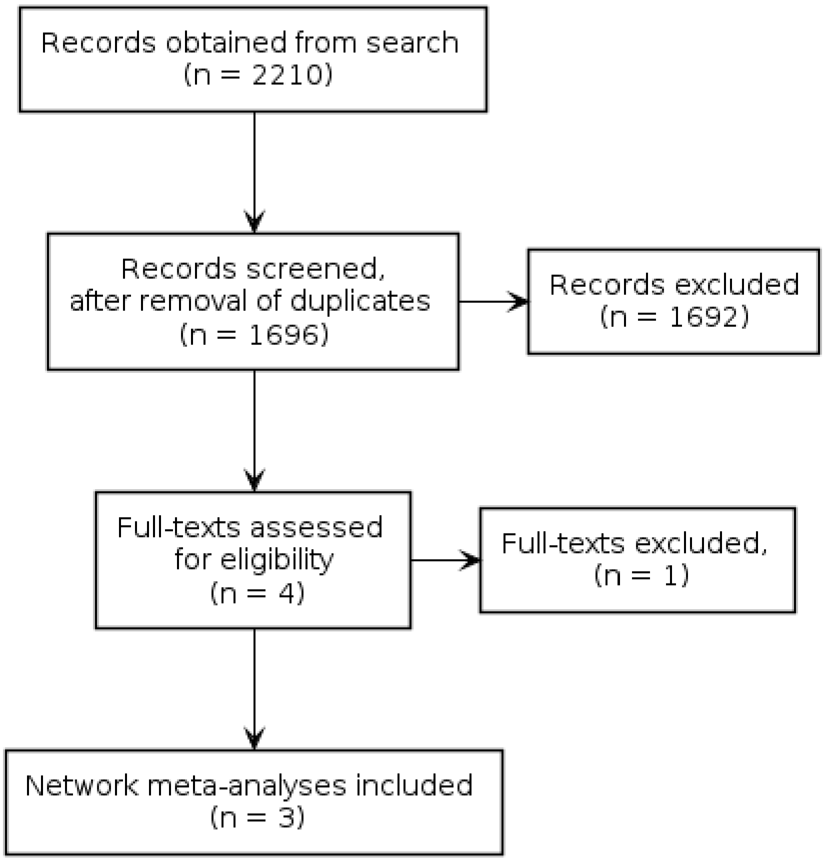

Table A.2: Trial characteristics

Study	Population	Intervention	Comparator	Included, N	Females, %	Age, mean (SD)	Fracture types	Trial registered	Trial registration discrepancies	RoB, functional outcomes/additional surgery
Boons 2012	Displaced 4-part PHFs including varus- and valgus-impacted fractures. Exclusion criteria: preexisting mental disorders, unable to provide informed consent or answer the questionnaires, disabling disorder or additional trauma to the affected arm, pathologic or open fracture, associated neurovascular injury, preexisting impairment of the contralateral shoulder, unable to understand the Dutch language, unable to participate in the rehabilitation protocol, and contraindication for surgery (ASA status I-III).	HA	NOP	50	94	78 (7.5)	Neer 4-part (100%)	No	—	H/M
Olerud 2011 HA	Acute displaced (≥10 mm and/or >45° angulation in relation to the head fragment, combined with a displacement of the greater and lesser tubercle of more than 10 mm in relation to the head fragment) 4-part PHFs in patients aged 55 years or older with a fracture sustained after a low-energy trauma with no previous shoulder problems, independent living conditions, no severe cognitive dysfunction, and with clearance by an anesthetist. Patients with a completely displaced shaft in relation to the head fragment were considered to have an absolute indication for surgery and were excluded. Patients with valgus-impaced fractures were also excluded.	HA	NOP	55	85	77 (NR)	Neer 4-part (100%)	No	—	H/M
Cai 2012	Acute displaced (≥10 mm and/or more than 45° of angulation in relation to the head fragment, combined with a displacement of the greater or lesser tubercle of more than	LCP	HA	32	84	72 (NR)	Neer 4-part (100%)	No	—	H/M

Study	Population	Intervention	Comparator	Included, N	Females, %	Age, mean (SD)	Fracture types	Trial registered	Trial registration discrepancies	RoB, functional outcomes/additional surgery
	10 mm in relation to the head fragment) 4-part PHFs in patients aged 67 years or older with a fracture sustained after a low-energy trauma, no previous shoulder problems, independent living conditions, and no severe cognitive dysfunction, and clearance by an anesthetist. Patients with a completely displaced shaft in relation to the head fragment were considered to have an absolute indication for surgery and were excluded. Patients with valgus-impaced fractures were also excluded.									
Chen 2016	Acute 4-part PHFs and/or fracture dislocations in patients with a mineral bone density less than –3.0. Exclusion criteria: previous history of shoulder surgery, chronic non-union, addiction to cigarettes and drugs, refusal to participate, or failure to cooperate.	LCP	HA	60	53	66 (NR)	Neer 4-part (100%)	No	—	H/H
Gracitelli 2016	Displaced (≥1 cm or ≥45° of angulation between the head and diaphysis of the humerus) PHFs, with or without involvement of the greater tuberosity, in patients aged between 50 and 85 years treated surgically ≤21 days after the injury. Exclusion criteria: isolated tuberosity fractures, articular split, fracture dislocation, open fractures, neurologic injury, previous surgery on the affected shoulder, associated fractures in the ipsilateral limb, pathologic fractures, psychiatric diseases, inability to understand the questionnaires, active or previous infection in the shoulder, irreparable tendon tears of the rotator cuff, and loss to follow-up before the first clinical assessment at 3 months.	LCP	IMN	65	72	65 (8.8)	Neer 2-part (49%) and 3-part surgical neck (51%)	Yes	Yes, registration states UCLA as outcome, but not reported.	H/H

Study	Population	Intervention	Comparator	Included, N	Females, %	Age, mean (SD)	Fracture types	Trial registered	Trial registration discrepancies	RoB, functional outcomes/additional surgery
Plath 2019	Acute isolated PHFs in patients aged 60 years or older with the capacity to provide informed consent. Exclusion criteria: isolated tuberosity fractures, previous trauma or surgery of the affected shoulder, advanced osteoarthritis, fracture dislocation, pathological fractures, open fractures, neurological disorders, full-thickness rotator cuff tears, and intra-operative change of treatment due to a fracture line through the nail entry point or where bone quality was considered not amenable to stable fixation with either implant.	LCP	IMN	68	75	74 (NR)	Neer 2-part (13%), 3-part (72%), and 4-part (15%)	No	—	H/H
Zhu 2011	Acute 2-part surgical neck fractures treated surgically within 21 days of injury in skeletally mature patients. Exclusion criteria: open physes, fracture and displacement involving the greater or lesser tuberosity or extension of the fracture line distally beyond the deltoid tubercle, associated musculoskeletal injuries to the ipsilateral upper extremity, an open fracture, and prior surgery on the affected shoulder.	LCP	IMN	51	67	53 (18.9)	Neer 2-part surgical neck (100%)	No	—	H/M
Fjalestad 2014	Displaced unstable 3- or 4-part PHFs (AO group 11-B2 or 11-C2, including subgroups 1,2 and 3 if fracture severely displaced: >45° angular deviation in true frontal or transthoracic radiographic projections regardless of whether or not the fracture was impacted, and greater or lesser tuberosity displaced at least 10 mm.) in patients aged 60 years or older. Exclusion criteria: displacement between the head and metaphyseal main fragments exceding 50% of the diaphyseal diameter, history of injury or illness of the injured or contralateral	LCP	NOP	50	88	73 (NR)	Neer 3-part (52%) 4-part (48%)	No	—	H/M

Study	Population	Intervention	Comparator	Included, N	Females, %	Age, mean (SD)	Fracture types	Trial registered	Trial registration discrepancies	RoB, functional outcomes/additional surgery
	shoulder, injuries of other parts of the humerus or the contralateral upper extremity, alcohol or drug abuse, dementia, neurologic diseases, severe cardiovascular diseases that would contraindicate surgery, and non-Scandinavian ethnicity.									
Launonen 2019	2-part low energy displaced PHFs where the fracture line emerges through the surgical (or anatomic) neck, in patients aged 60 years or older. Exclusion criteria: refusal to participate in the study, not independent, dementia and/or institutionalized, does not understand written and spoken guidance in either Finnish or Swedish, pathologic fracture or a previous fracture of the same proximal humerus, alcoholism or drug addiction, other injury to the same upper limb requiring surgery, major nerve injury (e.g., complete radial- or axillary nerve palsy), rotator cuff tear arthropathy, open fracture, multi-trauma or -fractured patient, fracture dislocation or head-splitting fracture, isolated fracture of the major or minor tubercle, gross displacement of the fracture fragments (no bony contact between fracture parts or the humerus shaft is in contact with the articular surface), any medical condition that excludes surgical treatment.	LCP	NOP	88	91	72 (7.6)	Neer 2-part surgical neck (100%)	Yes	Yes, reports OSS, but not mentioned in trial registration.	M/L
Olerud 2011 LCP	Acute displaced 3-part PHFs in patients aged 55 or older with a fracture sustained after a low-energy trauma, no previous shoulder problems, independent living conditions, no severe cognitive dysfunction, and with clearance by an anesthetist. Patients with a completely displaced shaft in relation to the head fragment were	LCP	NOP	59	81	74 (NR)	Neer 3-part (100%)	No	—	H/M

Study	Population	Intervention	Comparator	Included, N	Females, %	Age, mean (SD)	Fracture types	Trial registered	Trial registration discrepancies	RoB, functional outcomes/additional surgery
	considered to have an absolute indication for surgery and were excluded. Patients with valgus-impaced fractures were also excluded.									
Fraser 2020	Severly displaced (>45° valgus or >30° varus in a true anteroposterior projection, >45° angulation in a scapular Y projection with the arm in neutral rotation, or >50% displacement of the humeral head against the metaphysis) PHFs (AO type B2 or C2) in patients aged 65 to 85 years. Exclusion criteria: previous injury or illness of the injured or contralateral shoulder, concomitant injury to the ipsilateral or contralateral upper extremity, alcohol or other substance abuse, dementia or neurological disease, non-Norwegian-speaking patients, glenoid fracture or deformity, head-split fractures, fracture-dislocations, high-energy fractures, and patients deemed noncompliant to rehabilitation.	LCP	RSA	124	90	75 (6.4)	AO B2 (44%) and C2 (56%)	Yes	No	H/M
Sebastia-Forcada 2014	Acute PHFs with an indication for shoulder arthroplasty in consecutive patients aged 70 years or older. Indications for shoulder arthroplasty were complex fractures not amenable to reconstruction, including displaced 4-part fractures, fracture-dislocations with 3-part fractures, and head-splitting fractures with more than 40% articular surface involvement. Patients found intraoperatively to have irreparable cuff tears were not excluded. Exclusion criteria: contraindications to surgery, prior surgery in the shoulder, associated ipsilateral upper limb fracture, and neurologic disorder.	RSA	HA	61	85	74 (NR)	Neer 3-part (15%) and 4-part (85%)	No	—	M/M

Study	Population	Intervention	Comparator	Included, N	Females, %	Age, mean (SD)	Fracture types	Trial registered	Trial registration discrepancies	RoB, functional outcomes/additional surgery
Lopiz 2019	3- or 4-part displaced PHFs in patients aged 80 years or older who where available for follow-up for at least 12 months and able to understand the informed-consent process. Exclusion criteria: patients with mental disorders including cognitive impairment, open fracture, pathologic fracture, fracture-dislocation or head-splitting fracture according to Neer, neurologic disorder, associated ipsilateral or contralateral upper- or lower-limb fracture, prior surgery on the shoulder, or associated comorbidity contraindicating surgery, as well as patients who were not autonomous prior to the fracture as determined using the Katz index.	RSA	NOP	59	86	84 (5.1)	Neer 3-part (15%) and 4-part (85%)	No	—	H/M
Zyto 1997	Displaced 3- or 4-part PHFs with at least 30% contact between the humeral head and shaft. Exclusion criteria: high-energy trauma, pathological fractures, no other fractures elsewhere in the upper limbs, no concomitant disease likely to influence the end result, and inability of the patient to co-operate.	TB	NOP	40	88	74 (7.3)	Neer 3-part (92%) and 4-part (8%)	No	—	H/H

SD: standard deviation, RoB: risk of bias, HA: hemi-arthroplasty, NOP: nonoperative, LCP: locking compression plate, IMN: intramedullary nail, RSA: reverse shoulder arthroplasty, TB: tension-band, H: high, M: Moderate, L: Low, NR: Not reported, UCLA: University of California, Los Angeles score, OSS: Oxford shoulder score, PHF: proximal humerus fracture

Fig. A.2: Sensitivity analysis - network graphs

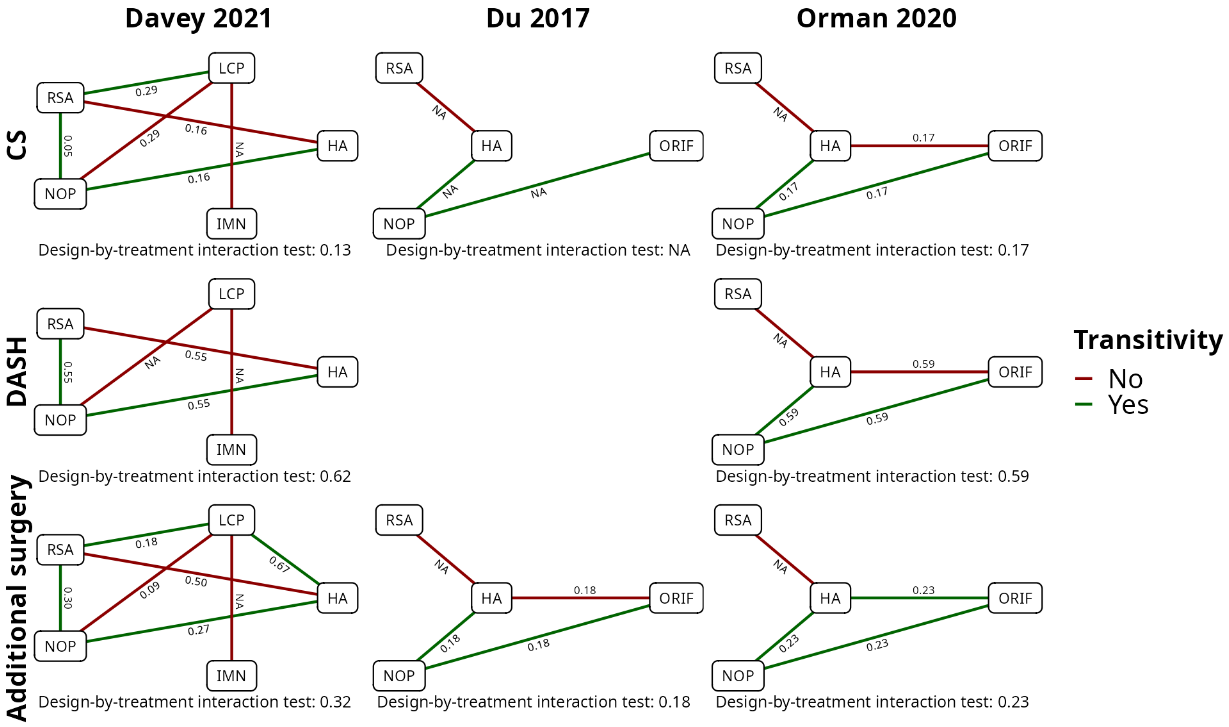

Fig.1: *Network graphs with transitivity and coherence for the included network meta-analyses. The numbers along the graph lines are the p-values obtained using SIDE (Separating Indirect from Direct Evidence) for that comparison. NA: not applicable due to lack of closed loop.*

Table A.3: Sensitivity analysis - CINeMA reasons for downgrading

Outcome domains	Studies, N	Comparisons, N	Within-study bias, %	Reporting bias, %	Intransitivity, %	Imprecision, %	Heterogeneity, %	Incoherence, %
**Davey 2021**								
CS	11	10	100	100	50	50	30	10
DASH	7	10	100	100	60	90	70	0
Additional surgery	11	10	100	100	10	60	30	10
**Du 2017**								
CS	6	6	100	100	33	50	50	100
Additional surgery	6	6	100	100	17	67	33	0
**Orman 2020**								
CS	7	6	100	100	67	50	67	0
DASH	4	6	100	100	83	NA	NA	NA
Additional surgery	7	6	100	100	67	67	33	0

CS: constant score, DASH: Disabilities of the Arm, Shoulder and Hand, NA: not applicable

Fig. A.3: Cai et al. 2012 sensitivity analysis - network graph

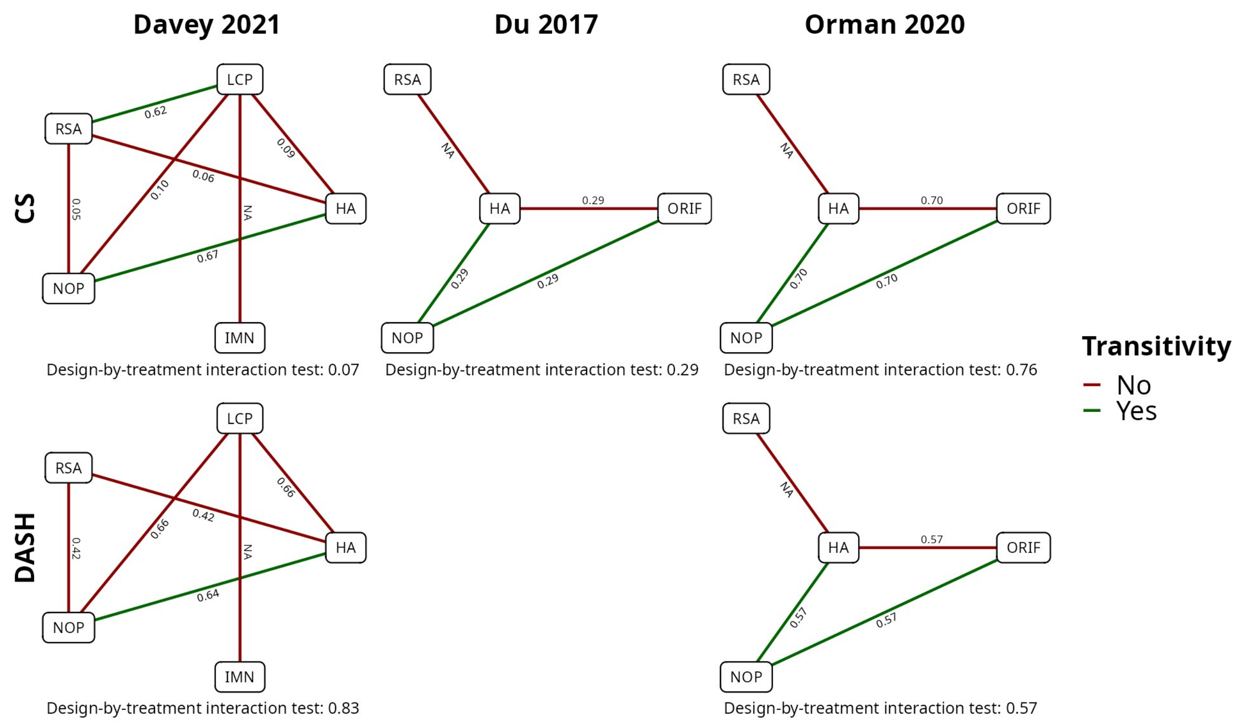

Fig.2: *Network graphs for CS and DASH using imputed SD for Cai et al. 2012. The numbers along the graph lines are the p-values obtained using SIDE (Separating Indirect from Direct Evidence) for that comparison. NA: not applicable due to lack of closed loop, SD: standard deviation.*

Table A.4: Cai et al. 2012 sensitivity analysis - CINeMA reasons for downgrading

Outcome domains	Studies, N	Comparisons, N	Within-study bias, %	Reporting bias, %	Intransitivity, %	Imprecision, %	Heterogeneity, %	Incoherence, %
**Davey 2021**								
CS	12	10	100	100	60	50	10	70
DASH	8	10	100	100	100	50	90	0
**Du 2017**								
CS	7	6	100	100	50	50	50	0
**Orman 2020**								
CS	8	6	100	100	67	50	50	0
DASH	5	6	100	100	83	33	100	0

CS: constant score, DASH: Disabilities of the Arm, Shoulder and Hand

Fig. A.4: Chen 2016 sensitivity analysis - network graph

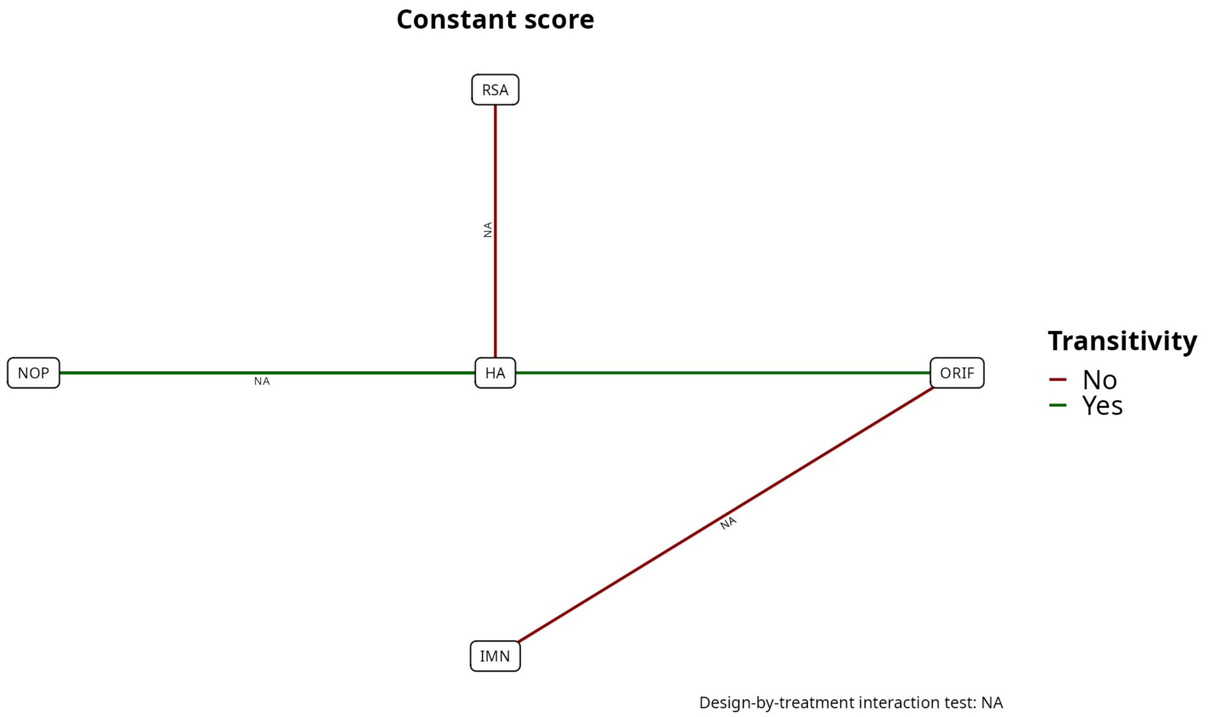

Fig.3: *Network graph for Chen 2016 with transitivity and coherence for the included network meta-analyses. The numbers along the graph lines are the p-values obtained using SIDE (Separating Indirect from Direct Evidence) for that comparison. NA: not applicable due to lack of closed loop.*

Table A.5: Chen 2016 sensitivity analysis - CINeMA reasons for downgrading

Outcome domains	Studies, N	Comparisons, N	Within-study bias, %	Reporting bias, %	Intransitivity, %	Imprecision, %	Heterogeneity, %	Incoherence, %
Constant score	8	10	100	100	60	50	40	100
